# First experiences with the Spectrum Compact CE System

**DOI:** 10.1007/s00414-021-02673-1

**Published:** 2021-10-19

**Authors:** Nastasja Burgardt, Melanie Weissenberger

**Affiliations:** Institute of Forensic and Traffic Medicine, Department of Forensic Genetics, University Hospital Heidelberg, Voßstraße 2, Building 4420, 69115 Heidelberg, Germany

**Keywords:** Spectrum Compact CE System, STR, Promega, DNA, Forensic genetics

## Abstract

The newly released Spectrum Compact CE System by Promega is a capillary electrophoresis instrument developed for DNA-fragment separation and sequencing. In this study, its compatibility to 8 commercial short tandem repeat (STR) kits from 4 different manufacturers, reproducibility (sizing precision, accuracy and concordance) and robustness (sensitivity and mixture resolution) were tested and compared to the ABI PRISM® 310 Genetic Analyzer. The instrument was able to successfully analyse amplicons of all tested kits, proved to be as precise as claimed by manufacturer specifications and was shown to be more robust than the ABI PRISM® 310 Genetic Analyzer in some aspects. Analyses on the Spectrum Compact CE System were able to resolve peaks with length differences of 1-basepair in the short and long fragment range and mixtures of mixture ratios down to 1:30. We describe the advantages and limitations we have observed so far working with this instrument in our forensic genetics laboratory.

**Supplementary Information:**

The online version contains supplementary material available at 10.1007/s00414-021-02673-1.

## Introduction

One of the most important steps of molecular genetic analysis is the separation and detection of DNA-fragments. Be it STR-analysis [1], sequencing [1], methylation analysis [2] or RNA-analysis [3], most molecular biology laboratories rely on some form of fragment separation to detect their analyte.

An effective way to achieve this separation is electrophoresis in combination with a sieving medium. In the beginning of DNA-profiling, slab-gel electrophoresis was used for that purpose [4]. But with the development of capillary electrophoresis (CE) separation methods in the early 1980s, research turned to applying CE to DNA analysis, since it requires less sample and solvent input and less run time, as well as offering a higher sample throughput and analysis sensitivity [5].

By 1988, commercial CE instruments started to be developed for a variety of applications [5]. The Dionex CESlA CE system (Dionex, Sunnyvale, CA, USA), the Applied Biosystems 270A-HT CE system (Applied Biosystems, Foster City, CA, USA) [6], the Beckman P/ACETM 2050 CE instrument (Beckman Instruments, Fullerton, CA, USA) [7, 8] and the Crystal CE System Model 310 (Unicam Corporation, Madison, WI, USA) [9], to name a few, were all tested and found to be suitable for application in Forensic DNA analysis. In 1996, Kuffner et al. [10] anticipated that evidence that has been analysed by capillary electrophoresis would soon be part of the judicial process and should be admissible (in US courts) since the method was, at that point, already widely accepted by the scientific community.

By the end of the 1990s, a CE system entered the market that was specialized for genotyping: the ABI PRISM® 310 Genetic Analyzer (Perkin Elmer, Applied Biosystems Division, Foster City, CA, USA) [11]. This single capillary instrument introduced the development and production of highly automated CE instruments for DNA analysis and is used in forensic genetics laboratories to this day.

**Fig. 1:**
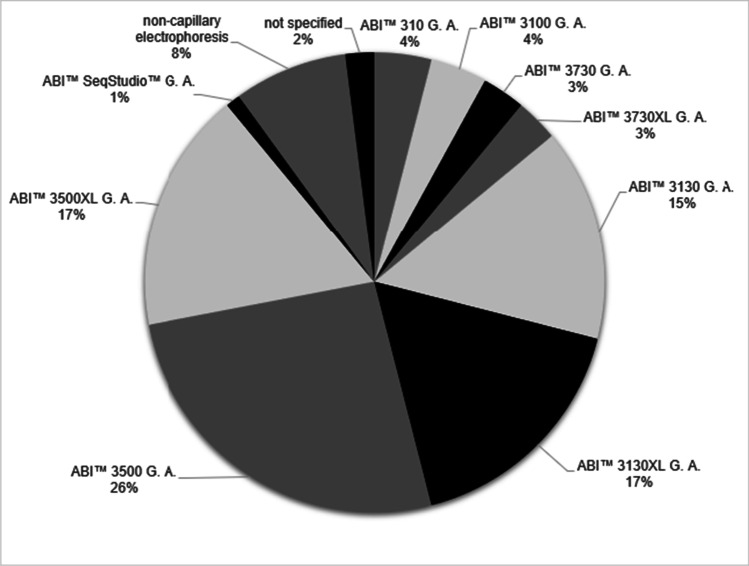
Pie chart of percentages of electrophoresis methods/instruments used in forensic genetics publications released going back 1 year from 18.01.2021 (*n* = 86) on https://pubmed.ncbi.nlm.nih.gov/, accessed under the search terms “forensic genetics DNA”, G.A., Genetic Analyzer

In the 86 publications that can be found under the search terms “forensic genetics DNA” on https://pubmed.ncbi.nlm.nih.gov/ [12] released during the last year (accessed on 18.01.2021) that made use of electrophoresis, the results published were achieved with the fragment separation methods shown in Fig. 1. The publications stem from 28 different journals and cover a range of topics including population genetics, application and validation of new techniques and multiplexes, animal and plant genetics, methylation, kinship testing, RNA analysis and identification cases.

While these results suggest a high number of instrument diversity, most of the laboratories used one of the two Applied Biosystems™ Genetic Analyzers 3130 or 3500 (both by Life Technologies Corporation, Carlsbad, CA, USA) with different capillary array numbers.

A monopoly on CE instruments for genetic purposes on the one hand means that comparability between laboratories is facilitated and that expertise and experience can be accrued and put into new products. But on the other hand, it can also create a dependency on a singular manufacturer and hinder diversity and innovation.

After two decades since the development of DNA analysis CE instrumentation, each laboratory that is looking to invest in a new CE instrument first has to establish its requirements for a new instrument.

The decision for an acquisition should be based on a variety of factors: sample throughput, usage frequency, budget (purchase as well as long-term cost), laboratory space, number and kind of different users and kits that are supposed to run on the instrument, etc.

It would therefore be of importance that the market for CE instruments for DNA analysis reflects the diversity of laboratory needs.

This publication is based on the acquisition of a CE instrument as a replacement of/supplement to the 1-capillary ABI PRISM® 310 Genetic Analyzer.

The replacement instrument requirements were as follows:

- comparable sample throughput to the ABI PRISM® 310 Genetic Analyzer
- preferably new/with a recent release as to avoid a discontinuation of manufacturer support in the near future
- compatibility with the pre-established analysis software (GenoProof Mixture 2 by qualitype GmbH, Dresden, Germany)
- compatibility to kits of different suppliers (including 6-dye kits)
- economical solutions for consumables (no hard-stops, easy changing, etc.)
- equal or better analysis quality compared to the ABI PRISM® 310 Genetic Analyzer
- easily understandable user interface for fast training of new laboratory personnel

Since there are currently no other 1-capillary CE instruments available, the choice fell on a 4-capillary instrument, excluding instruments with higher sample throughput like the Applied Biosystems™ Genetic Analyzer 3500 since our laboratory requires smaller injection sizes.

The Spectrum Compact CE System was newly released in 2020 as the first CE instrument by Promega developed in collaboration with Hitachi High-Tech [13].

Among the beforementioned requirements, the goal of this publication was to share the first experiences with the Spectrum Compact CE System in a forensic genetics laboratory and compare it to the ABI PRISM® 310 Genetic Analyzer.

## Materials and methods

### Instruments and consumables

The instrument features and information about consumables for the ABI PRISM® 310 Genetic Analyzer and the Spectrum Compact CE System are shown in Table 1.

**Table 1:** Instrument features of the ABI PRISM® 310 Genetic Analyzer and the Spectrum Compact CE System in comparison

	ABI PRISM® 310 Genetic Analyzer	Spectrum Compact CE System
Capillary number/array	1	4
Capillary length	47 cm	36 cm
Possible dye channel colours	Up to 5	Up to 6
Polymer for fragment analysis	POP4 (in this study)	Spectrum Compact Polymer4
Laser/wavelength	10-mW argon ion class 1 laser (when safety interlocks are enabled, otherwise class 3b), 488 nm and 514 nm	20-mW class 1 laser, 505 nm
Detection unit	CCD camera	CCD camera
Sample tray	48 single sample tubes (in this study)	4 strips × 8 sample wells = 32 samples
Control system	External computer required	All system functions can be controlled at the instrument touchscreen itself, external computer required for data analysis
Output data format for fragment analysis	.fsa-files	.fsa-files
Data connection	Automatic transfer of.fsa-files to connected computer	Instrument has its own network connection, download of.fsa-files by remote software from any device that is connected to the network possible, as well as directly from the instrument (USB port at the front of the instrument)
Quality indicators calculated by instrument	None	Spectral calibration: Quality value and condition number Fragment analysis: Sizing quality and electrophoresis quality
Runtime (including washing and filling capillary steps)	35 min/sample	42 min/4 samples
Size and weight	61 cm × 55.9 cm × 86.4 cm, 90 kg	40 cm × 60 cm × 60 cm, 45 kg
Power input	240 Vac; 50/60 Hz; 2.4 kVA	100–240Vac; 50/60 Hz; 260VA
Operating temperature	15 to 30 °C	15 to 30 °C
Soft stops	Warning when there is not enough polymer left for the planned run setup Warning when the capillary exceeded its expiry (after 100 injections)	Warning anode buffer (number of usage/expiry) Warning cathode buffer (number of usage/expiry) Warning capillary array (number of usage/expiry) Warning polymer (expiry date)
Hard stops	None (except when polymer is empty, if the warning was ignored, the instrument stops in the middle of the run when polymer is empty)	When polymer is empty or is less than required for the planned run set up, the run cannot be started

The capillary of the ABI PRISM® 310 Genetic Analyzer was within its recommended number of injections, but other capillaries have regularly been used beyond expiry (up to 2000 injections). The capillary-array in the Spectrum Compact CE System used in this publication has been in use for over 5 months with a break of 3 weeks in between when it was removed from the instrument and was used up to 200 injections (100%) beyond the recommended injection number.

The quality indicators given by the Spectrum Compact CE System were checked for each spectral calibration (quality value QV and condition number CN) and each fragment analysis (sizing quality SQ and electrophoresis quality EQ).

All runs were performed at an environmental temperature of 25 °C (air condition controlled).

### Spectral calibration and compatibility

#### ABI PRISM® 310 Genetic Analyzer

For the analysis with the three STR-kits PowerPlex® ESX17, PowerPlex® Y23 (Promega, Fitchburg, WI, USA) and Investigator® Argus X-12 QS (Qiagen, Hilden, Germany), the colour filters G5v2 (for PowerPlex® ESX17 and PowerPlex® Y23) and G5 (for Investigator® Argus X-12 QS) were used for the spectral calibration, as recommended by the manufacturers.

Each colour has been run separately and combined to a matrix according to the instruction of the manufacturers.

#### Spectrum Compact CE System

The spectral calibration was performed using the pre-installed assays (Table 2) on the Spectrum Compact CE System for the Spectrum Compact Polymer4. For each matrix, a dye set containing all colours of the STR kit was run on the four capillaries.

**Table 2:** STR-kits with corresponding pre-installed filter assay colour sets/run-assays that were used on the Spectrum Compact CE System

Kit	Assay	Kit dyes	Filter assay colours
MPX5ESSv5	Filter6_5Dye_O600(60–600)_36_P4	FAM, JOE-C6, YEL, RED & ORN	6-FAM™, VIC®, NED™, PET™ & LIZ™
NGM Detect™	T_6Dye_O600(60–600)_36_P4	6-FAM™, VIC™, TED™, TAZ™, SID™ & LIZ™	6-FAM™, VIC™, NED™, TAZ™, SID™, LIZ™
PowerPlex® ESX17 & PowerPlex® Y23	Promega_5Dye_WENILS_36_P4	Fluorescein, JOE, TMR-ET, CXR-ET & WEN	Fluorescein, JOE, TMR-ET, CXR-ET & WEN
Investigator® Argus X-12 QS, Investigator® ESSplex SE QS & Investigator® IDPlex Plus	Q_5Dye_BTO550_36_P4	6-FAM™, BTG, BTY, BTR & BTO	6-FAM™, BTG, BTY, BTR & BTO
Investigator® 24plex QS	Q_6Dye_BTO550_36_P4	6-FAM™, BTG, BTY, BTR2, BTP & BTO	6-FAM™, BTG, BTY, BTR2, BTP & BTO

Eight STR-genotyping kits (PowerPlex® ESX17, PowerPlex® Y23, Investigator® Argus X-12 QS, Investigator® 24plex QS (Qiagen, Hilden, Germany), Investigator® ESSplex SE QS (Qiagen, Hilden, Germany), Investigator® IDPlex Plus (Qiagen, Hilden, Germany), MPX5ESSv5 (Serac, Bad Homburg, Germany) and NGM Detect™ (Applied Biosystems™, Foster City, CA, USA) by four different suppliers were tested (matrix installation followed by a few sample test-runs, run conditions are shown in Table 3) to assess the flexibility of the instrument.

**Table 3:** Run conditions used on the ABI PRISM® 310 Genetic Analyzer and Spectrum Compact CE System

Instrument	Injection time	Injection voltage	Run voltage	Run temperature	Run time
ABI PRISM® 310 Genetic Analyzer	5 s	15 kV	15 kV	60 °C	28 min
Spectrum Compact CE System	9 s	1.6 kV	13 kV	60 °C	32.17 min

### Precision, accuracy and concordance

To determine the precision of the Spectrum Compact CE System, two injections (*n* = 8) of the PowerPlex® ESX17 allelic ladder were analysed and compared between and within the injections by standard deviation (SD).

To determine the accuracy of the Spectrum Compact CE System, *n* = 27 sample-runs (13 1-person-samples and 14 mixtures in 8 injections) were compared to one PowerPlex® ESX17 allelic ladder (injected as first on capillary 1) by mean base-pair (bp) deviation.

A total number of 109 samples with known results (known control alleles or comparison to ABI PRISM® 310 Genetic Analyzer results) were analysed with 6 different autosomal and 2 gonosomal kits to check for concordance.

### Baselines and carry-over

To determine the noise-level of analyses of the Spectrum Compact CE System for the kits PowerPlex® ESX17, PowerPlex® Y23, Investigator® Argus X-12 QS and NGM Detect™, the mean peak height of *n* = 10 amplification negative controls per kit (analysed with the analytical threshold of 1 rfu) was calculated.

Based on these values, the limits of detection (LOD) and limits of quantitation (LOQ) were calculated separately for each colour channel by adding either 3 or 10 SD to the mean.

The same was done for PowerPlex® ESX17, PowerPlex® Y23 and Investigator® Argus X-12 QS amplicons on the ABI PRISM® 310 Genetic Analyzer for comparison.

A high amount of DNA (~ 4.73 ng DNA PCR-input) was analysed on the Spectrum Compact CE System to test for carry-over to the next injection (negative control).

### Dynamic range with thresholds

To determine the dynamic range and analysis thresholds of the Spectrum Compact CE System, a dilution series of the positive amplification control 2800 M (Promega) was amplified in duplicate with the PowerPlex® ESX17 kit and each sample was then analysed in quadruplicate (*n* = 76).

The 750 pg-level was not amplified in duplicate as it was added at the second amplification.

The 10 calculated dilution levels were as follows:

2ng, 1 ng, 750 pg, 500 pg, 250 pg, 125 pg, 62.5 pg, 31.25 pg, 15.63 pg and 7.81 pg

The dilution series was set up based on the concentration given on the amplification control (10 ng/µL). 2800 M concentration is measured by the manufacturer by PowerQuant® Real-Time Assay analysis with the specifications of an acceptable range of 9–11 ng/uL and an A260/280 ratio of > 1.75 [14].

Concentrations were confirmed by measurements with the QuantiFluor® dsDNA system on a Quantus fluorometer (both by Promega) down to 62.5 pg. Below that dilution, measurement data became unreliable and DNA amounts are given in approximate calculated values.

The limits of linearity (LOL) were determined separately for each colour channel.

The stochastic threshold was defined by the mean of all heterozygous peaks at DNA template input levels where drop-outs occur plus 3 SD [15].

The limit of linearity was defined as the highest measured on-scale (without flat-tops or splits) allele peak in a heterozygous locus.

The LOL was then combined with the LOD and the LOQ to create an overview over the range, in which a linear relationship between signal and DNA-input amount can be expected (dynamic range of the Spectrum Compact CE System).

A replicate of all amplicons was analysed on the ABI PRISM® 310 Genetic Analyzer for comparison.

Allele calls were made based on the LODs determined in section “Baselines and carry-over”.

### 1-bp resolution

To assess the 1-bp resolution of the Spectrum Compact CE System, the peak-to-valley ratio (valley height divided by the smaller peak height) of alleles that differ in 1-bp in D1S1656 was calculated with *n* = 29 (2 amplicons of the Gednap 47 trace 4 sample amplified with PowerPlex® ESX17 and 5 PowerPlex® ESX17 allelic ladders) for the shorter fragment region (155–170 bp) and *n* = 24 (2 amplicons of the Gednap 47 trace 4 sample amplified with NGM Detect™ and 5 NGM Detect™ allelic ladders) for the longer fragment region (290–360 bp).

This was compared to the peak-to-valley ratios of 1 amplicon of the Gednap 47 trace 4 sample amplified with PowerPlex® ESX17 and 5 PowerPlex® ESX17 allelic ladders (*n* = 27) run on the ABI PRISM® 310 Genetic Analyzer.

### Mixture resolution

A mixture of the positive amplification controls 2800 M (PowerPlex® ESX17 kit control) and DNA Control 007 (NGM Detect™ kit control) in five mixture ratios (1:1, 1:5, 1:10, 1:20, 1:30) was amplified in duplicate with the PowerPlex® ESX17 kit and analysed in duplicate on the Spectrum Compact CE System.

The same amplicons were analysed on the ABI PRISM® 310 Genetic Analyzer for comparison.

The calculated DNA template amounts chosen for the mixture of 2800 M (major contributor) and DNA Control 007 (minor contributor) were as follows:

- 250 pg + 250 pg for the 1:1 mixture
- 500 pg + 100 pg for the 5:1 mixture
- 750 pg + 75 pg for the 10:1 mixture
- 1 ng + 50 pg for the 20:1 mixture
- and 1 ng + 33 pg for the 30:1 mixture.

One nanogram was chosen as the maximum DNA template input as the Spectrum Compact CE System showed to reach its limit of linearity at that DNA template amount.

The mixtures were set up based on the concentration given on the amplification controls (10 ng/µL on 2800 M and 0.1 ng on DNA Control 007). The concentrations of the positive controls were confirmed by measurements with the QuantiFluor® dsDNA system on a Quantus fluorometer.

### Colour channel balance, heterozygote ratios and stutter ratios

The colour channel balances and stutter incidences/ratios for the kits PowerPlex® ESX17, PowerPlex® Y23, Investigator® Argus X-12 QS and NGM Detect™, as well as the heterozygote ratios for the kits PowerPlex® ESX17, Investigator® Argus X-12 QS and NGM Detect™ were calculated based on 1-person profiles with ideal DNA template input of ~ 250 pg (*n* = 17 per instrument per kit for the colour channel balances and heterozygote ratios and *n* = 10 per instrument per kit for the stutter ratios). NGM Detect™ data was only generated on the Spectrum Compact CE System, since the ABI PRISM® 310 Genetic Analyzer cannot support 6 colour dye kits.

### Sample analysis

#### Samples, extractions and quantification

The samples used for this study were Gednap proficiency test samples, DGAB (Deutsche Gesellschaft für Abstammungsbegutachtung) proficiency test samples, positive controls from commercial PCR-kits and known samples of individuals who have given their written consent to use their buccal swab DNA extracts for scientific purposes according to §13 of the German law of genetic diagnostics. The sample extractions were done using either a Chelex-extraction [16] or the GEN-IAL® First-DNA all tissue kit (GEN-IAL GmbH, Troisdorf, Germany).

All DNA samples (except for the mixtures) and positive controls were quantified by measuring 2 μL of each extract (with a second confirmation measurement that had to lie between ± 0.03 ng/μL of the original measurement) using the QuantiFluor® dsDNA system on a Quantus fluorometer. The samples were then (if needed) diluted for the PCR according to those measurements.

#### PCR, CE and data analysis

All PCR amplifications were carried out on a Biometra Trio 30 (Analytik Jena, Jena, Germany). For the autosomal Qiagen kits (Investigator® ESSplex SE QS, Investigator® IDPlex Plus and Investigator® 24plex QS), the full recommended reaction volume of 25 μL per sample was used since these kits were not previously validated in our laboratory. All other kits were applied with a reduced reaction volume of 12.5 μL per sample.

For injection on the Spectrum Compact CE System, 1 μL of PCR product was combined with the internal size standard according to the manufacturer of the STR-kit and 9.5 μL Hi-Di™-formamide (Applied Biosystems™) in MicroAmp™ Optical 8-Tube Strips (0.2 mL) (Applied Biosystems™). The strips were covered with “Strip Septa Mat, 8-Well” strip septa mats (Promega) and the contents were vortexed, spun down, denatured at 95 °C for 3 min and then snap-cooled on a cold block for a minimum of 2 min. The sample strips were set up using Spectrum Compact Control Software and then loaded onto the Spectrum Compact CE System.

All PCR products were separated by capillary electrophoresis on the Spectrum Compact CE System using a 36-cm 4-capillary array filled with Spectrum Compact Polymer4 (Promega).

The comparison samples were analysed on the ABI PRISM® 310 Genetic Analyzer according to manufacturer guidelines.

The analysis of the.fsa data was carried out with the GenoProof Mixture 2 software (qualitype GmbH, Dresden, Germany).

#### Run conditions

The run conditions on the ABI PRISM® 310 Genetic Analyzer and the Spectrum Compact CE System are shown in Table 3.

## Results

### Instruments and consumables

All run quality indicators (sizing quality and electrophoresis quality) on the Spectrum Compact CE System were always passed, except for the MPX5ESSv5 runs. Despite the failed quality indicators, interpretable and correct electropherograms were produced (example supplemental Fig. 6 in “Online Resource 1”).

With 200 more injections than the recommended 200, the EQ values given by the Spectrum Compact CE System of the capillary used in this study are still satisfactory for analysis (meaning that the EQ values of the analyses are ≥ 400).

### Spectral calibration and compatibility

The quality values and condition numbers calculated by the Spectrum Compact CE System for its spectral calibrations for five different matrices (Promega 5-dye, T6dye, Q5dye, Q6dye and Filter6 5dye), as well as the minimum quality value and the maximum condition number that is used by the instrument to decide between fail and pass, are shown in Table 4.

**Table 4:** Spectral calibration parameter pass conditions and measured values for all four capillaries of the capillary-array of the Spectrum Compact CE System

	Min. quality value	Max. condition number	Capillary number	Quality value measured	Condition number measured
Promega 5-DYE	≥ 0.95	≤ 8.5	1	0.996	5.66
			2	0.996	5.81
			3	0.995	5.67
			4	0.997	5.54
T6dye	≥ 0.95	≤ 8.0	1	0.995	5.91
			2	0.991	6.30
			3	0.994	6.05
			4	0.998	5.90
Q5dye	≥ 0.95	≤ 20	1	0.994	6.84
			2	0.994	6.78
			3	0.996	6.75
			4	0.994	6.73
Q6dye	≥ 0.95	≤ 13.5	1	0.993	11.19
			2	0.993	11.23
			3	0.991	11.16
			4	0.996	11.08
Filter6 5dye	≥ 0.95	≤ 13.5	1	0.979	6.13
			2	0.997	6.83
			3	0.998	6.71
			4	0.997	6.62

Example electropherograms of amplifications with MPX5ESSv5, NGM Detect™, PowerPlex® ESX17, PowerPlex® Y23, Investigator® Argus X-12 QS, Investigator® ESSplex SE QS, Investigator® IDPlex Plus and Investigator® 24plex QS are shown in “Online Resource 1” (supplemental Figs. 6–13).

### Precision, accuracy and concordance

The highest SD calculated for the Spectrum Compact CE System based on *n* = 8 PowerPlex® ESX17 ladders was 0.07 bp. There was no significant difference in deviation between and within the injections.

In a run of 27 PowerPlex® ESX17 amplicons (8 injections), the mean deviation of the samples from the allelic ladder (injection 1, capillary 1) was 0.10 bp. The highest deviation was 0.38 bp in the locus D18S51 (injection 7, capillary 3).

A total of 109 samples were tested with the Spectrum Compact CE System with 6 different autosomal and 2 gonosomal kits and were all concordant with the expected results.

### Baselines and carry-over

The calculated LODs and LOQs (rounded to the closest 5-value) for the Kits PowerPlex® ESX17, PowerPlex® Y23 and Investigator® Argus X-12 QS for both instruments and for the kit NGM Detect™ on the Spectrum Compact CE System are shown in Table 5 and supplemental Table 1 in “Online Resource 1”.

**Table 5:** Limits of detection (LOD) and limits of quantitation (LOQ) on the Spectrum Compact CE System (*n* = 40) for the kits PowerPlex® ESX17, PowerPlex® Y23, Investigator® Argus X-12 QS and NGM Detect™, rounded to the closest 5-value (rfu)

	LOD (rfu) Spectrum Compact CE System					LOQ (rfu) Spectrum Compact CE System				
Colour channel	Blue	Green	Yellow	Red	Violet	Blue	Green	Yellow	Red	Violet
PowerPlex® ESX17	25	20	20	35	n/a	60	40	50	75	n/a
PowerPlex® Y23	25	25	20	50	n/a	50	55	55	105	n/a
Investigator® Argus X-12 QS	35	20	20	55	n/a	75	40	45	120	n/a
NGM Detect™	30	30	35	40	30	50	50	75	65	55

The negative control that was run on the Spectrum Compact CE System on the same capillary after an amplicon of ~ 4.73 ng DNA showed no carry-over.

### Dynamic range and thresholds

#### Dilution series

At 2 ng DNA template input, off-scale peaks and pull-ups occurred on both instruments and in both amplicons.

At 1 ng DNA template input on the Spectrum Compact CE System, off-scale peaks occurred mostly in one amplicon, while in the other, only the single homozygous system in the yellow channel (D22S1045 allele 16 at ~ 101.5 bp) and therefore its neighbouring allele 15 in system D10S1248 (at ~ 101.5 bp) in the green channel were partially off-scale because of the spectral overlap. At 1 ng DNA template input on the ABI PRISM® 310 Genetic Analyzer, off-scale peaks and strong pull-ups occurred in both amplicons.

At 750 pg DNA template input on the Spectrum Compact CE System, off-scale peaks only occurred in the single homozygous system (D22S1045) and allele 15 in D10S1248 (see to 1 ng). In the single 750 pg analysis performed on the ABI PRISM® 310 Genetic Analyzer, off-scale peaks occurred in D22S1045 and D12S391 and strong pull-ups throughout all colour channels.

At 500 pg DNA template input on the Spectrum Compact CE System, only the single homozygous system (D22S1045) and allele 15 in D10S1248 (see to 1 ng) showed off-scale peaks, while off-scale peaks in D2S441 and D12S391 and small pull-ups were observed on the ABI PRISM® 310 Genetic Analyzer.

At 250 pg DNA template input, both instruments produced full on-scale profiles without pull-ups.

At 125 pg DNA template input, the heterozygote ratio fell below 60% in 6 of the systems on both instruments, with the lowest observed heterozygote ratio observed being at around 32% in D8S1179 on both instruments. Both instruments produced full on-scale profiles.

At 62.5 pg DNA template input, full on-scale profiles with heterozygote ratios of down to around 30% are produced on both instruments.

At ~ 31 pg DNA template input, full on-scale profiles with heterozygote ratios of down to 10% are produced on both instruments.

At ~ 16 pg DNA template input on the Spectrum Compact CE System, first full and partial drop-outs occurred (6% of expected alleles missing) with the tallest false homozygote observed at 421 rfu in the D16S539 system. On the ABI PRISM® 310 Genetic Analyzer, full and partial dropouts (9% of expected alleles missing) were observed, with the tallest false homozygote being 296 rfu in the D16S539 system.

At ~ 8 pg DNA template input on the Spectrum Compact CE System, full and partial drop-outs (29% of expected alleles missing) were observed. On the ABI PRISM® 310 Genetic Analyzer, full and partial drop-outs occurred (33% of expected alleles missing).

#### Stochastic threshold

Based on the mean of all heterozygous peaks at DNA template input levels where drop-outs occur (~ 16 pg and ~ 8 pg) plus 3 SD, the stochastic thresholds for the PowerPlex® ESX17 kit were determined as 424 rfu on the ABI PRISM® 310 Genetic Analyzer and 670 rfu on the Spectrum Compact CE System.

#### Dynamic range and limit of linearity

The highest on-scale peaks of the dilution series analysed on the Spectrum Compact CE System were observed at 1 ng DNA template input at 32,560 rfu in the blue colour channel, 26,242 rfu in the green colour channel, 31,699 rfu in the yellow colour channel and 29,898 rfu in the red colour channel. The LOL was therefore reached at 1 ng DNA template input, since no linear signal/template relationship was observed after this point.

The highest on-scale peaks of the dilution series analysed on the ABI PRISM® 310 Genetic Analyzer were observed at 7444 rfu in the blue colour channel (at 750 pg DNA template input), 7289 rfu in the green colour channel (at 500 pg DNA template input), 6986 rfu in the yellow colour channel (at 2 ng DNA template input) and 7137 rfu in the red colour channel (at 750 pg DNA template input). The LOL was reached at 500 pg DNA template input, since no linear signal/template relationship was found after this point, except for in the yellow colour channel, where signal strengths were lower and the linearity extends up to 750 pg.

**Fig. 2:**
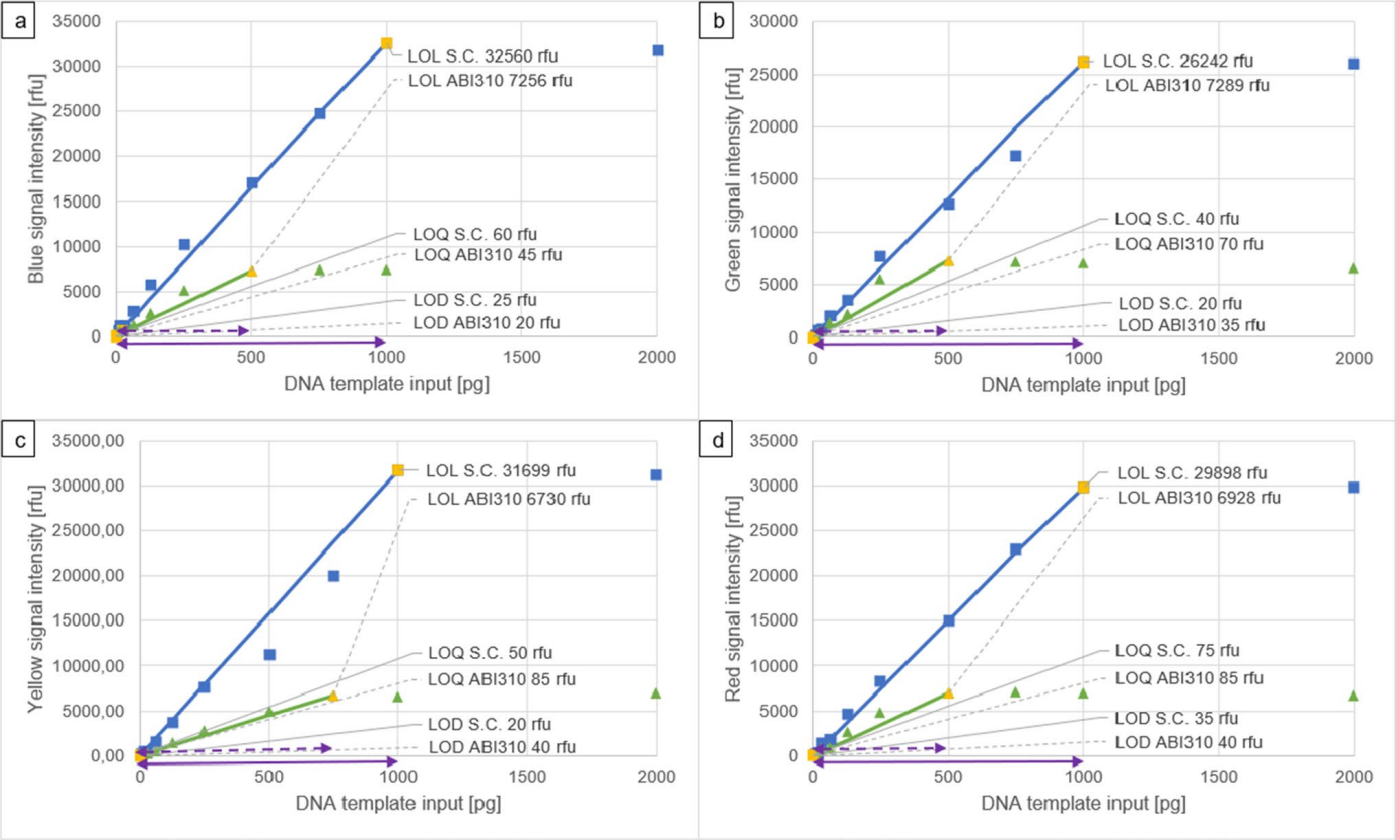
Dynamic ranges of the Spectrum Compact (S.C.) CE System (continuous arrows) and the ABI Prism® 310 (ABI310) Genetic Analyzer (dashed arrows) with the limit of detection (LOD), limit of quantitation (LOQ) and limit of linearity (LOL) thresholds for each instrument for each dye of the PowerPlex® ESX17 kit in relation to the maximum on-scale peak signals per DNA template input amount, (*n* = 76 for each colour on the Spectrum Compact CE System, *n* = 19 for each colour on the ABI Prism® 310 Genetic Analyzer); **a** blue colour channel; **b** green colour channel; **c** yellow colour channel; **d** red colour channel

The dynamic ranges of the Spectrum Compact CE System and the ABI Prism® 310 Genetic Analyzer of the four colour channels based on the baseline and dilution series analysis of the PowerPlex® ESX17 amplicons are shown in Fig. 2.

### 1-bp resolution

#### ABI Prism® 310 Genetic Analyzer

Allelic ladder peak analysis on the ABI PRISM® 310 Genetic Analyzer showed an average peak-to-valley value of 44.7% (SD 5.66%) with the PowerPlex® ESX17 kit at the locus D1S1656 (150–175 bp).

**Fig. 3:**
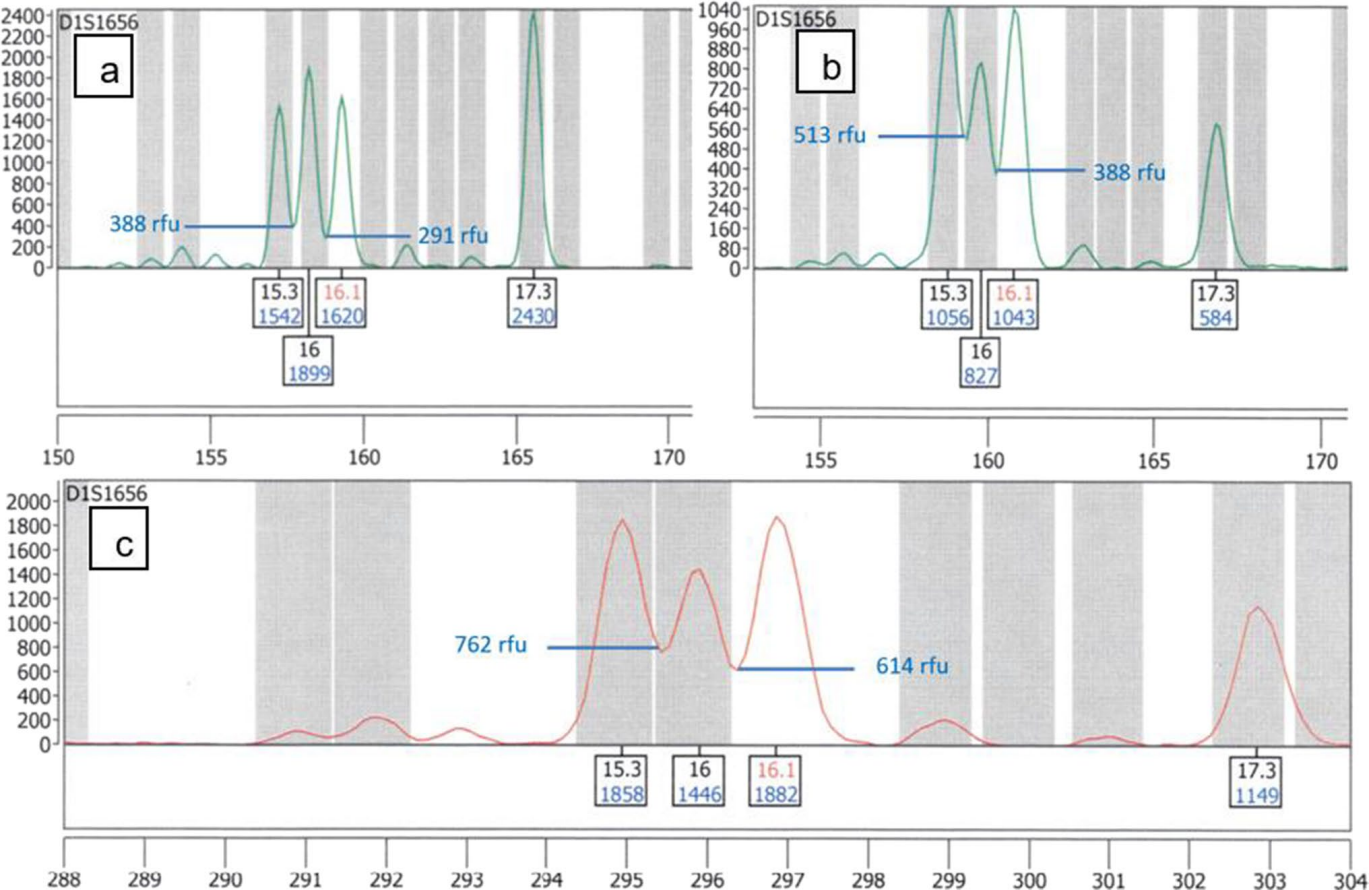
Electropherograms of Gednap 47 trace 4 sample in the locus D1S1656 with indicated valley heights; **a** PowerPlex® ESX17 on the Spectrum Compact CE System, amplicon 1; **b** PowerPlex® ESX17 on the ABI PRISM® 310 Genetic Analyzer; **c** NGM Detect™ on the Spectrum Compact CE System, amplicon 2

The calculated peak-to valley values for the Gednap 47 trace 4 sample in the locus D1S1656 was 62% and 46.9% (Fig. 3b).

#### Spectrum Compact CE System

The peak-to-valley values in the shorter fragment length range (150–175 bp) of allelic ladder peaks on average were 17.65% (SD 2.25%) (PowerPlex® ESX17) and 59.92% (SD 4.65%) in the longer fragment length range (NGM Detect™, 290–310 bp).

The calculated peak-to valley values for the Gednap 47 trace 4 sample in the locus D1S1656 were 25% and 17.9% for amplicon 1 (Fig. 3a) and 24% and 18.8% for amplicon 2 (PowerPlex® ESX17) in the shorter fragment length region and 65% and 40% for amplicon 1 and 52.6% and 42.5% for amplicon 2 (Fig. 3c) (NGM Detect™) in the longer fragment length region.

### Mixture resolution

Complete interpretable mixture profiles were achieved down to a ratio of 1:10 (75 pg + 750 pg) with the Spectrum Compact CE System and down to a ratio of 1:5 (100 pg + 500 pg) with the ABI Prism® 310 Genetic Analyzer.

In the following, exemplary loci for different major/minor constellations will be shown.

#### Minor and major heterozygous, no shared alleles

Mixture loci in which the minor as well as the major were heterozygous and shared no alleles which were analysed on the Spectrum Compact CE System were all readable down to a mixture ratio of 1:30 (except for one dropout incident at 1:30 in D18S51 in one replicate).

**Fig. 4:**
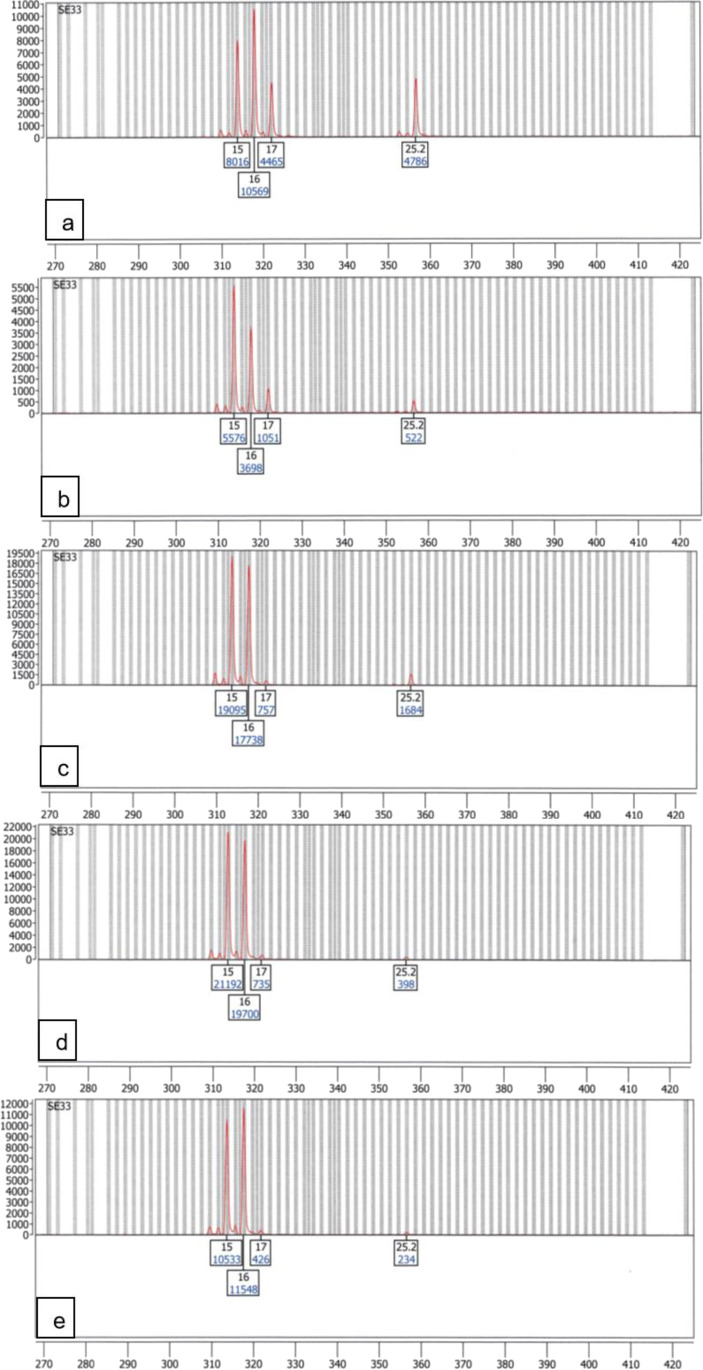
Electropherograms of a mixture of the two DNA amplification positive controls 2800 M and DNA Control 007 in 5 mixture ratios at the locus SE33 (minor alleles 17/25.2 and major alleles 15/16), amplified with the PowerPlex® ESX17 kit and analysed on the Spectrum Compact CE System, only true alleles are labelled; **a** mixture ratio 1:1; **b** mixture ratio 5:1; **c** mixture ratio 10:1; **d** mixture ratio 20:1; **e** mixture ratio 30:1

The mixture analysis can be seen represented by the locus SE33 in Fig. 4. While the Spectrum Compact CE System analyses show a clear and calculable (1:33.46, Fig. 4e) mixture profile in the lowest mixture ratio, the ABI PRISM® 310 Genetic Analyzer analyses reached their limit of evaluability at the 1:10 ratio (75 pg + 750 pg), where the ABI PRISM® 310 Genetic Analyzer exceeds its limit of linearity and produces off-scale peaks and pull-ups (supplemental Fig. 1 in “Online Resource 1”).

#### Minor and major heterozygous, one shared allele

Mixture loci in which the minor and the major were heterozygous and shared one allele which were analysed on the Spectrum Compact CE System were readable down to a mixture ratio of 1:30.

**Fig. 5:**
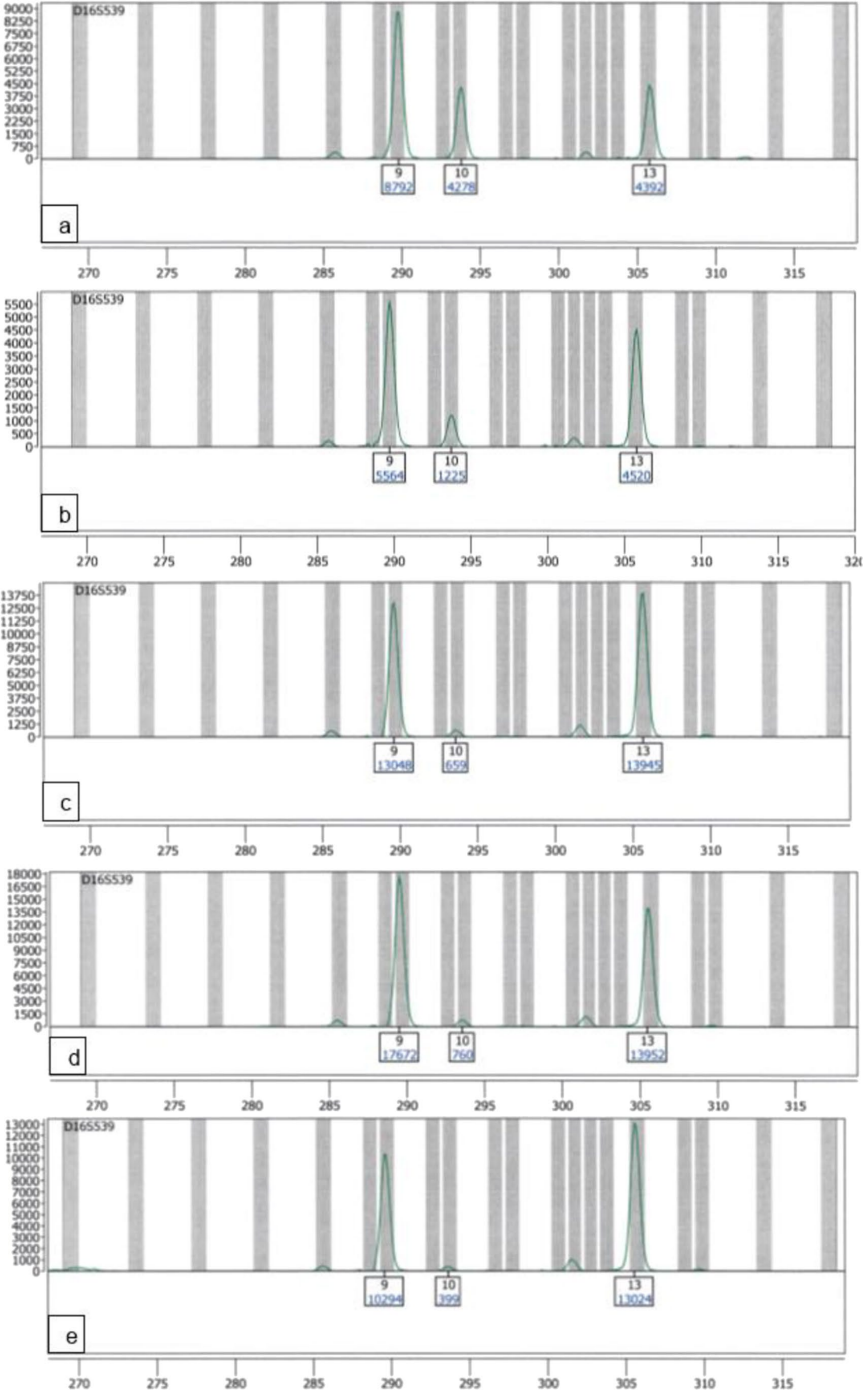
Electropherograms of a mixture of the two DNA amplification positive controls 2800 M and DNA Control 007 in 5 mixture ratios at the locus D16S539 (minor alleles 9/10 and major alleles 9/13) amplified with the PowerPlex® ESX17 kit and analysed on the Spectrum Compact CE System, only true alleles are labelled; **a** mixture ratio 1:1; **b** mixture ratio 5:1; **c** mixture ratio 10:1; **d** mixture ratio 20:1; **e** mixture ratio 30:1

The mixture analysis analysed on the Spectrum Compact CE System can be seen represented by the locus D16S539 in Fig. 5. While the Spectrum Compact CE System analyses show a clear and readable mixture profile in the lowest mixture ratio (Fig. 5e), the ABI PRISM® 310 Genetic Analyzer analyses reached their limit of evaluability at the 1:20 (50 pg + 1 ng) ratio, where the ABI PRISM® 310 Genetic Analyzer exceeds its limit of linearity and produces off-scale peaks and pull-ups (supplemental Fig. 2 in “Online Resource 1”).

#### Minor heterozygous, major homozygous, one shared allele

Mixture loci in which the minor was heterozygous, the major was homozygous and they shared one allele which were analysed on the Spectrum Compact CE System were readable down to a mixture ratio of 1:10.

**Fig. 6:**
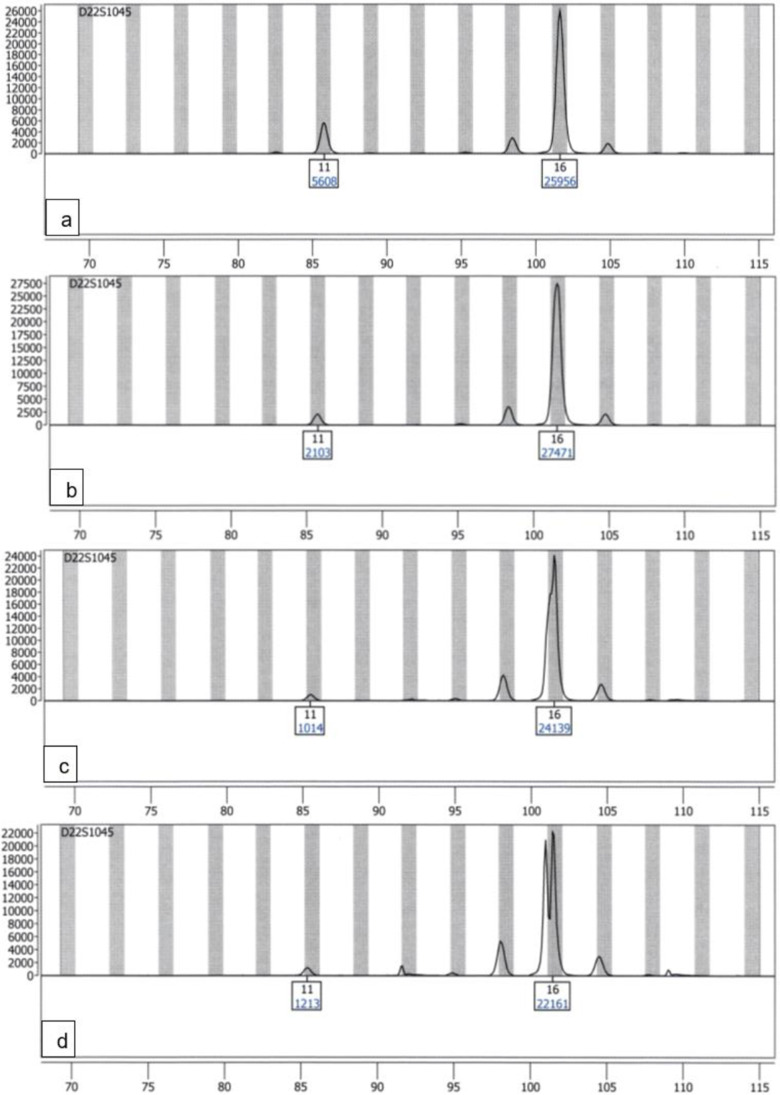
Electropherograms of a mixture of the two DNA amplification positive controls 2800 M and DNA Control 007 in 4 mixture ratios at the locus D22S1045 (minor alleles 11/16 and major alleles 16/16), amplified with the PowerPlex® ESX17 kit and analysed on the Spectrum Compact CE System, only true alleles are labelled; **a** mixture ratio 1:1; **b** mixture ratio 5:1; **c** mixture ratio 10:1; **d** mixture ratio 20:1, only true alleles are labelled, unlabelled peaks that are not in stutter-positions correspond to pull-ups from other systems

The mixture analysis can be seen represented by the locus D22S1045 in Fig. 6. While the Spectrum Compact CE System analyses show a clear mixture profile (Fig. 6c) in the 1:10 mixture ratio (at which the limit of linearity was reached), the ABI PRISM® 310 Genetic Analyzer analyses reached their evaluability limit at the 1:10 (75 pg + 750 pg) ratio, where the ABI PRISM® 310 Genetic Analyzer exceeds its limit of linearity and produces strong pull-ups (supplemental Fig. 3 in “Online Resource 1”).

### Colour channel balance, heterozygote ratios and stutter

#### Colour channel balance

With the PowerPlex® ESX17 kit, all colour channels performed within ± 5% around their ideal percentage except for the green colour channel (JOE, 6% deviation) on the Spectrum Compact CE System and the red colour channel (CXR-ET, 7% deviation) on the ABI PRISM® 310 Genetic Analyzer.

With the PowerPlex® Y23 kit, all colour channels performed within ± 5% around their ideal percentage except for the blue colour channel (Fluorescein, 6% deviation) and the red colour channel (CXR-ET, 7% deviation) on the ABI PRISM® 310 Genetic Analyzer.

With the Investigator® Argus X-12 QS kit, all colour channels performed within ± 5% around their ideal percentage except for the blue colour channel (6-FAM™, 9% deviation) on the Spectrum Compact CE System.

With the NGM Detect™ kit, the colour channels of the Spectrum Compact CE System perform less proportionately. The green colour channel (VIC™) deviates by 8% and the yellow colour channel (TED™) by 9% from their ideal percentage.

**Fig. 7:**
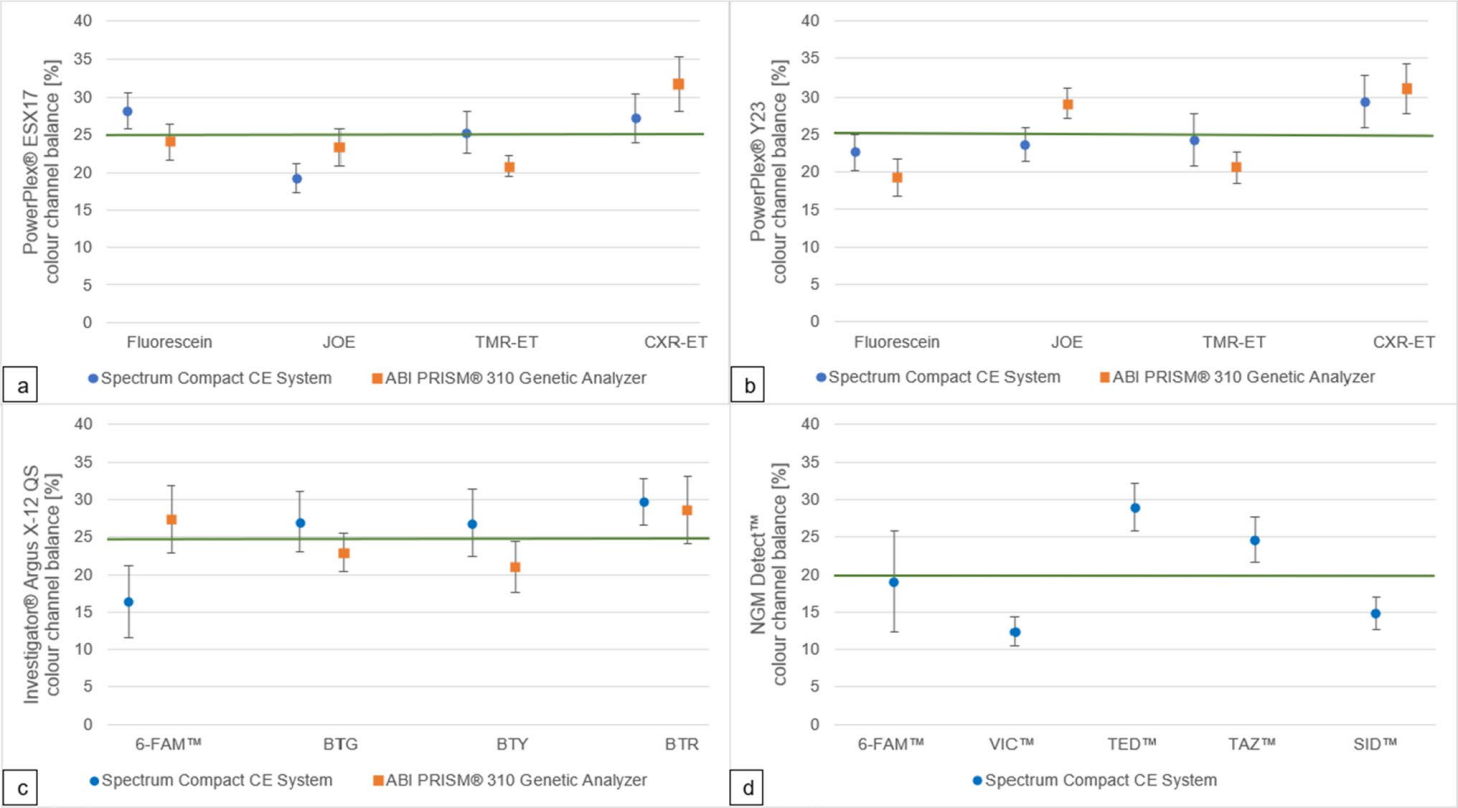
Colour channel balances (%) on the Spectrum Compact CE System and the ABI PRISM® 310 Genetic Analyzer based on *n* = 17 samples per instrument per kit with the ideal percentage share indicated by a thick line; **a** PowerPlex® ESX17; **b** PowerPlex® Y23; **c** Investigator® Argus X-12 QS; **d** NGM Detect™

An overview of the channel balances for the kits PowerPlex® ESX17, PowerPlex® Y23 and Investigator® Argus X-12 QS on the ABI PRISM® 310 Genetic Analyzer and Spectrum Compact CE System, as well as NGM Detect™ on the Spectrum Compact CE System, is shown in Fig. 7.

#### Heterozygote ratios

The heterozygote ratios for an ideal DNA template input of ~ 250 pg on both instruments with the kits PowerPlex® ESX17 and Investigator® Argus X-12 QS, as well as NGM Detect™ on the Spectrum Compact CE System were comparable and were mainly above 60% (supplemental Table 2 in “Online Resource 1”). Outliers of down to 46% with the PowerPlex® ESX17, 32% with the NGM Detect™ and 50%/30% (ABI PRISM® 310 Genetic Analyzer/Spectrum Compact CE System) with the Investigator® Argus X-12 QS kit have been observed.

#### Stutter ratios

Analyses on the Spectrum Compact CE System showed comparable stutter ratios to the ABI PRISM® 310 Genetic Analyzer (examples for the PowerPlex® ESX17 kit are shown in supplemental Figs. 4 and 5 in “Online Resource 1”).

## Discussion: Advantages and limitations of the Spectrum Compact CE System

### Instruments and consumables

The Spectrum Compact CE System is a relatively small CE instrument, in actual size as well as in its capacity. With its 4-capillary array and 32-sample tray capacity it is not suitable for laboratories which require a higher sample throughput. When sample numbers that are not divisible by four are run, more consumables are wasted than they would have been with a single capillary instrument. For a small laboratory with a corresponding sample volume, however, a 4-capillary instrument serves as a good compromise when a single-capillary system is no longer available.

The Spectrum Compact CE System can analyse 6 dye channels, which is an improvement compared to the ABI PRISM® 310 Genetic Analyzer and therefore allows the implementation of 6-dye kits like the NGM Detect™.

The kits used in this study, like most commercially available STR kits, have been developed and validated by the manufacturers with polymer 4, which is the polymer used for fragment analysis on the Spectrum Compact CE System. This facilitates the use of commercially available kits on the instrument, since one parameter less needs to be adjusted for.

Internal quality checks by the Spectrum Compact CE System were all passed and conclusive with the observable quality of the results. The calculation of SQ (which is “determined by comparing the fragment pattern observed for the size standard being used against that specified for the size standard in the Sizecalling Protocol” [13]) and the EQ value (“size in bases at which the peak width at half maximal height is equal to the distance between two bases as calculated from the size standard” [13]) was not possible for the MPX5ESSv5 amplicons because there is no specific pre-installed sizing information available on the instrument. A solution for lacking pre-installed data might be found through a software update.

The user interface is intuitive and straightforward and therefore easily learnable. The flexible run set up that can be done on either the touch screen of the instrument or the companion software, which facilitates re-runs and strip-setup corrections. The interface has a panel for consumables that gives a direct overview over the consumables and clearly indicates warnings before and when consumables should be replaced.

The Spectrum Compact CE System has no hard stops, which allows the user to monitor the quality of the analyses at their own discretion if they want to continue using consumables, like the capillary array or the buffers, beyond their recommended injection number. This can save a lot of resources, as demonstrated with the capillary array used in this study which already has been in use for 200% of its recommended 200 injections. This advantage is comparable to the capillary use of the ABI PRISM® 310 Genetic Analyzer, where the capillary could be used beyond its recommended injection number (up to 2000 injections) without loss of analysis quality. Capillary quality can be monitored by observing the peak properties of the analyses which is facilitated by the EQ values given out by the Spectrum Compact CE System.

All consumables are separately integrated in the instrument and can be exchanged or taken out and stored, if the instrument is temporarily not in use. The 2D barcodes on the consumables are scanned by a hand scanner before they get installed in the instrument, so the information about each individual consumable (charge number, injections left, storage life, etc.) is tracked by the instrument. This means the consumption of polymers, buffers and capillaries is not connected, but depends on the actual usage of each. Stored consumables which are re-installed into the instrument can be recognized by the scanner and the information about whether the consumable should still be used and how many injections it has left is available again. If the 2D barcodes are or become unreadable, they can easily be substituted by scanning separate print-outs of the barcodes of the same lot.

Since the consumables are designed for flexible exchange, their size is intended for fewer injections than the consumables of larger, less flexible instruments. A single polymer cartridge lasts for 64 samples/16 injections and the buffer cartridges for 320 samples/80 injections. The user will therefore, depending on the analysis frequency, have to replace/reorder them accordingly.

There are no special installation requirements beyond the space needed and a connection to a normal power outlet.

Runs can only be started when there is enough polymer left for the planned run set-up.

The instrument is capable of Sanger-sequencing, but this has yet to be tried by our laboratory.

### Spectral calibration and compatibility

The Spectrum Compact CE System calculates two parameters for the spectral calibration for each capillary in the array. The quality value “describes the confidence with which fluorescent signal from any given dye can be separated from that contributed by the other fluorescent dyes present. The highest theoretical value is 1.0, with no signal from any given fluorescent dye contributing to signal from any of the other fluorescent dyes” [13]. The condition number is a “measure of the degree to which there is overlap in the spectral emission profiles of the dyes used in a given dye set” [13]. Its ideal value would be 1.0.

All spectral calibrations performed were successful as judged by the parameter passes, even though not all matrixes corresponded specifically to a pre-programmed filter assay. The matrix installations for a selection of 8 STR kits from 4 different suppliers that are used in forensic DNA analysis were successful and easily executed because of the pre-programmed spectral calibration programmes. The instrument assays cover a wide range of commonly used commercial STR kits and can be adapted to uncommon or self-made kits using the “Filter” settings, as can be seen with the spectral calibration of the MPX5ESSv5 kit. The instrument compatibility with kits from different manufacturers makes it possible to perform confirmation analyses with kits from different manufacturers on the same instrument.

The spectral calibration of the ABI PRISM® 310 Genetic Analyzer on the other hand is a lot more complex and time consuming, since every colour of the matrix has to be run separately and then combined.

All tested kits produced readable electropherograms with peaks of similar heights which lie between the stochastic threshold and the LOL (except for Investigator® IDPlex Plus in the system TH01), affirming their compatibility with the Spectrum Compact CE System. All colours were differentiated without overlap.

An inter-locus imbalance was noticeable for the kits Investigator® ESSplex SE QS, Investigator® IDPlex Plus and Investigator® 24plex QS, which have been applied with a DNA input amount of ~ 500 pg and a reaction volume of 25 µL as recommended by the manufacturer, since their PCR-conditions have not previously been optimized in our laboratory. These imbalances could be improved by optimizing the PCR conditions and are not dependant on the instrument the fragment separation was performed on.

### Precision, accuracy and concordance

The sizing precision value determined in this study concurs with the value of 0.07 bp supplied by Promega [17]. Since the sizing precision was shown to be the same between and within injections, data that was generated using different capillaries and different injections can be confidently compared and analysed with ladders from other injections from the run.

The accuracy of the instrument showed that run conditions inside the Spectrum Compact CE System stay stable enough so that only one allelic ladder is necessary per maximum 31 samples (a run equals max. 8 injections x 4 samples including at least one ladder).

A new ladder injection every 10 samples was necessary when working with the ABI PRISM® 310 Genetic Analyzer, but as opposed to the Spectrum Compact CE System, one allelic ladder tube can be reinjected by itself several times for an analysis of up to 47 samples in one run.

All samples analysed which had known results were concordant with known control DNA alleles and/or the results of the ABI PRISM® 310 Genetic Analyzer.

### Baselines and carry-over

The limits of detection which were determined based on the baseline of the Spectrum Compact CE System were comparable to those of the ABI PRISM® 310 Genetic Analyzer. The LODs set based on the baseline analysis were applied separately for each colour channel in this study. If a single threshold had been set as the highest LOD (35 rfu), 4 allele calls (1.7% of all expected alleles) would have been missed in the PowerPlex® ESX17 dilution series on the Spectrum Compact CE System.

On the ABI PRISM® 310 Genetic Analyzer, 3.3% of allele calls would have been lost with a universal threshold of 40 rfu, supporting the colour channel specific threshold approach.

No carry-over has been observed in any negative controls.

### Dynamic range with thresholds

The dynamic range of the Spectrum Compact CE System goes up to twice the DNA template input amount of what the ABI PRISM® 310 Genetic Analyzer is able to analyse (1 ng as opposed to 500 pg). For the Spectrum Compact CE System, the theoretical cut-off of 32,767 rfu was almost reached (99.37%) by the highest observed on-scale peak of 32,560 rfu in the blue channel at 1 ng DNA template input with the PowerPlex® ESX17 kit.

On the ABI PRISM® 310 Genetic Analyzer, the highest observed on-scale peak of 7444 rfu in the blue channel at 750 pg DNA template input with the PowerPlex® ESX17 kit came close to the theoretical cut-off of 8192 rfu (90.87%).

The higher dynamic range of the Spectrum Compact CE System allows for the analysis of highly degraded DNA samples.

At low DNA template input amounts, the sensitivity of the two instruments is comparable, making the analysis of low template DNA samples on the Spectrum Compact CE System possible.

The stochastic thresholds were set using the mean of all heterozygous peaks at DNA template input levels where drop-outs occur (~ 16 pg and ~ 8 pg) plus 3 SD which includes all tallest false homozygotes found in this study. The stochastic threshold determined for the Spectrum Compact CE System was higher than the one determined for the ABI PRISM® 310 Genetic Analyzer, which is consistent with the findings that the Spectrum Compact CE System produces higher signal peaks at the same DNA template input amounts.

### 1-bp resolution

The Spectrum Compact CE System showed better 1-bp resolution than the ABI PRISM® 310 Genetic Analyzer and was able to provide clear peak resolution even in the longer fragment range.

### Mixture resolution

Due to the higher dynamic range of the Spectrum Compact CE System, mixtures of higher contributor DNA amount differences can be analysed than on the ABI PRISM® 310 Genetic Analyzer since higher amounts of DNA template input are possible without overloading the detection unit.

The overall interpretation of mixture profiles could be aided by secondary analysis with kits that have a different locus distribution, as to differentiate real peaks from pull-ups.

### Colour channel balance, heterozygote ratios and stutter

The colour channel balance between the two instruments is comparable, with the red colour channel being stronger than the other channels if CXR-ET is analysed on the ABI PRISM® 310 Genetic Analyzer and 6-Fam™ being weaker in the Investigator® Argus X-12 QS kit on the Spectrum Compact CE System.

The NGM Detect™ seems to be less balanced than the other kits, but that could stem from the spectral calibration assay being configured for a NED™ yellow dye and not a TED™ as is used in the kit.

Heterozygote ratios and stutter ratios are comparable on both instruments and no influence beyond PCR-factors was noticed.

### Sample analysis

The sample throughput of 4 samples/42 min is higher than on the ABI PRISM® 310 Genetic Analyzer (roughly 1 sample/35 min).

To analyse samples on the Spectrum Compact CE System that have been amplified by a kit for which the instrument has pre-installed run assays, no parameter adjustments are necessary.

On the ABI PRISM® 310 Genetic Analyzer, each run parameter has to be selected manually if more than one kit requiring different run parameters is part of one run, while the run parameters on the Spectrum Compact CE System are integrated in the specific run modules of each kit. This facilitates the run-setup as it is less time consuming and less prone to human error.

The.fsa files generated were compatible for analysis with the GenoProof Mixture 2 software.

## Conclusion

The Spectrum Compact CE System meets the requirements for forensic DNA analysis and of a laboratory with a high diversity of samples (e.g. low template DNA, degraded DNA, mixtures with a strong major/weak minor) and times of usage.

We assess that the Spectrum Compact CE System is a suitable tool for DNA-fragment separation and will continue to gather data about our experience working with it.

## Availability of data

Not applicable.

## Supplementary Information

Below is the link to the electronic supplementary material.

Supplementary file1 (DOCX 7721 kb)

## Data Availability

Not applicable.
