# Supplementary material for: First experiences with the Spectrum Compact CE System
Source: Int J Legal Med. 2021 Oct 19;136(1):55–72. doi: 10.1007/s00414-021-02673-1 (PMC8813819; doi:10.1007/s00414-021-02673-1)
Supplement: Supplementary file 1 — Supplementary file1 (DOCX 7721 kb) [file 414_2021_2673_MOESM1_ESM.docx]

**First experiences with the Spectrum Compact CE System – Online Resource 1**

*International Journal of Legal Medicine*

Nastasja Burgardt^a^ (CA^b^) & Melanie Weissenberger^a,c^

**Online Resource 1**

Supplemental data with information regarding the ABI PRISM® 310 Genetic Analyzer, PCR-dependant comparison data and exemplary electropherograms.

^a^ Institute of Legal Medicine and Traffic Medicine, Department of Forensic Genetics, University Hospital Heidelberg, Voßstraße 2, Building 4420, 69115 Heidelberg, Germany

^b^ corresponding author: nastasja.burgardt@med.uni-heidelberg.de; Institut für Rechts- und Verkehrsmedizin, Voßstraße 2, Gebäude 4420, 69115 Heidelberg, Germany;

^b^ ORCID iD 0000-0002-0162-6844

^c^ [melanie.weissenberger@med.uni-heidelberg.de](mailto:melanie.weissenberger@med.uni-heidelberg.de), ORCID iD 0000-0002-9305-3077

|  | LOD [rfu] ABI PRISM® 310 Genetic Analyzer | | | | LOQ [rfu] ABI PRISM® 310 Genetic Analyzer | | | |
| --- | --- | --- | --- | --- | --- | --- | --- | --- |
| Colour channel | Blue | Green | Yellow | Red | Blue | Green | Yellow | Red |
| PowerPlex® ESX17 | 20 | 35 | 40 | 40 | 45 | 70 | 85 | 85 |
| PowerPlex® Y23 | 20 | 35 | 35 | 35 | 50 | 75 | 75 | 75 |
| Investigator® Argus X-12 QS | 20 | 25 | 45 | 40 | 40 | 55 | 95 | 90 |

**Supplemental table 1** Limits of detection (LOD) and limits of quantitation (LOQ) [rfu] on the ABI PRISM® 310 Genetic Analyzer (n=30) for the kits PowerPlex® ESX17, PowerPlex® Y23 and Investigator® Argus X-12 QS, rounded to the closest 5-value


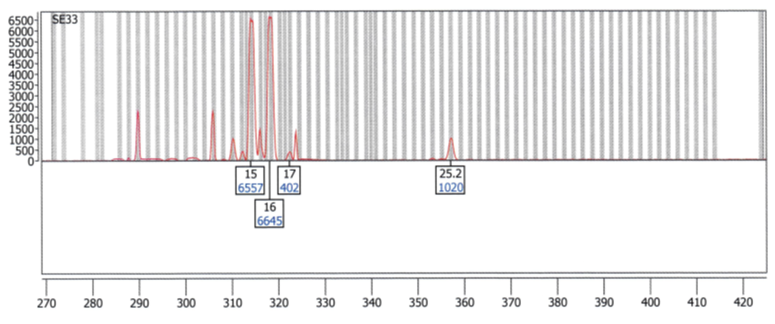


**Supplemental figure 1** Electropherogram of a mixture of the two DNA amplification positive controls 2800M and DNA Control 007 in the mixture ratio 1:10 at the locus SE33 (minor alleles 17/25.2 and major alleles 15/16) amplified with the PowerPlex® ESX17 kit and analysed on ABI PRISM® 310 Genetic Analyzer, only true alleles are labelled, unlabelled peaks that are not in stutter-positions correspond to pull-ups from other systems


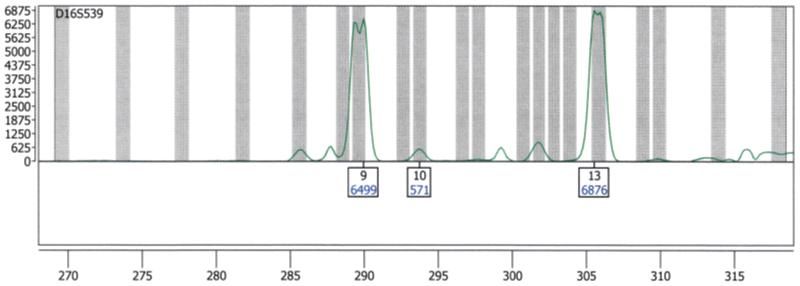


**Supplemental figure 2** Electropherogram of a mixture of the two DNA amplification positive controls 2800M and DNA Control 007 in the mixture ratio 1:20 at the locus D16S539 (minor alleles 9/10 and major alleles 9/13), amplified with the PowerPlex® ESX17 kit and analysed on ABI PRISM® 310 Genetic Analyzer, only true alleles labelled, unlabelled peaks that are not in stutter-positions correspond to pull-ups from other systems


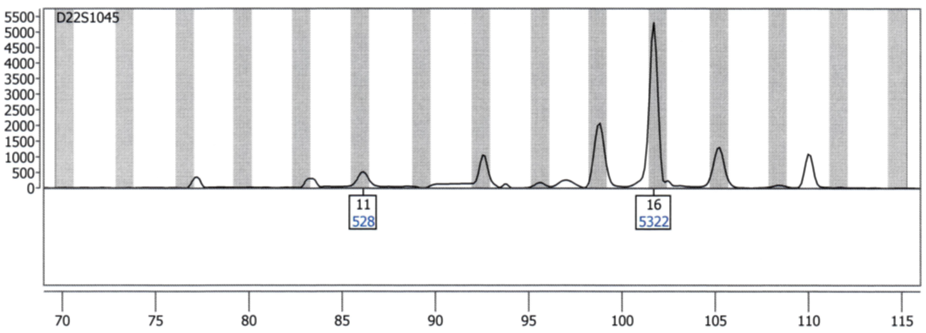


**Supplemental figure 3** Electropherograms of a mixture of the two DNA amplification positive controls 2800M and DNA Control 007 in the mixture ratio 1:10 at the locus D22S1045 (minor alleles 11/16 and major alleles 16/16), amplified with the PowerPlex® ESX17 kit and analysed on ABI PRISM® 310 Genetic Analyzer, only true alleles are labelled, unlabelled peaks that are not in stutter-positions correspond to pull-ups from other systems

|  | ABI PRISM® 310 Genetic Analyzer | | Spectrum Compact CE System | |
| --- | --- | --- | --- | --- |
|  | mean | SD | mean | SD |
| PowerPlex® ESX17 | 86.36 % | 10.13 % | 83.87 % | 10.61 % |
| Investigator® Argus X-12 QS | 80.63 % | 13.06 % | 83.93 % | 11.27 % |
| NGM Detect™ | n/a | n/a | 84.17 % | 11.50 % |

**Supplemental table 2** Mean heterozygote-ratios [%] with SD of the kits PowerPlex® ESX17and Investigator® Argus X-12 QS on the ABI PRISM® 310 Genetic Analyzer and Spectrum Compact CE System and NGM Detect™ on the Spectrum Compact CE System, n=17 per kit per instrument


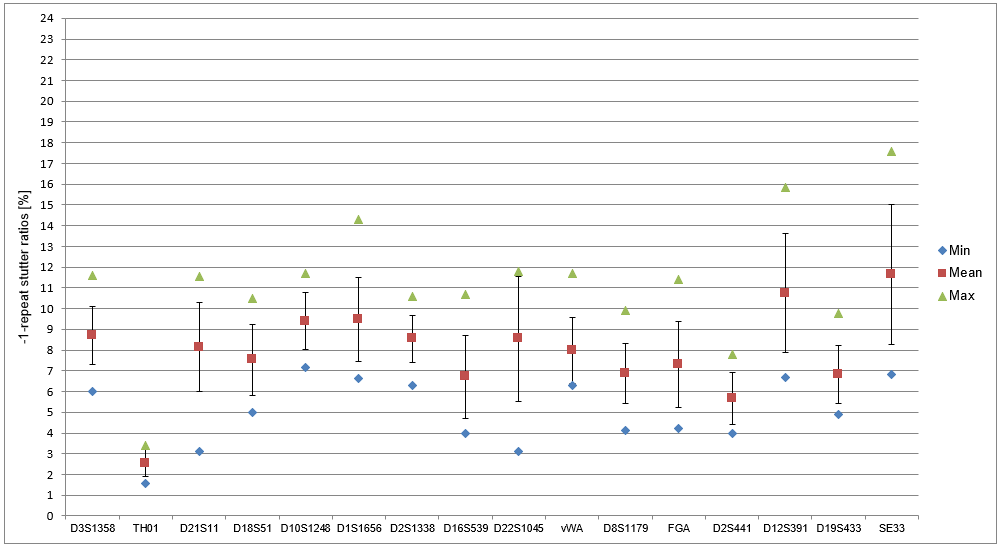


**Supplemental figure 4** -1-repeat stutter ratios [%] (minima, mean with SD and maxima) of the PowerPlex® ESX17 kit on the Spectrum Compact CE System (n=10)


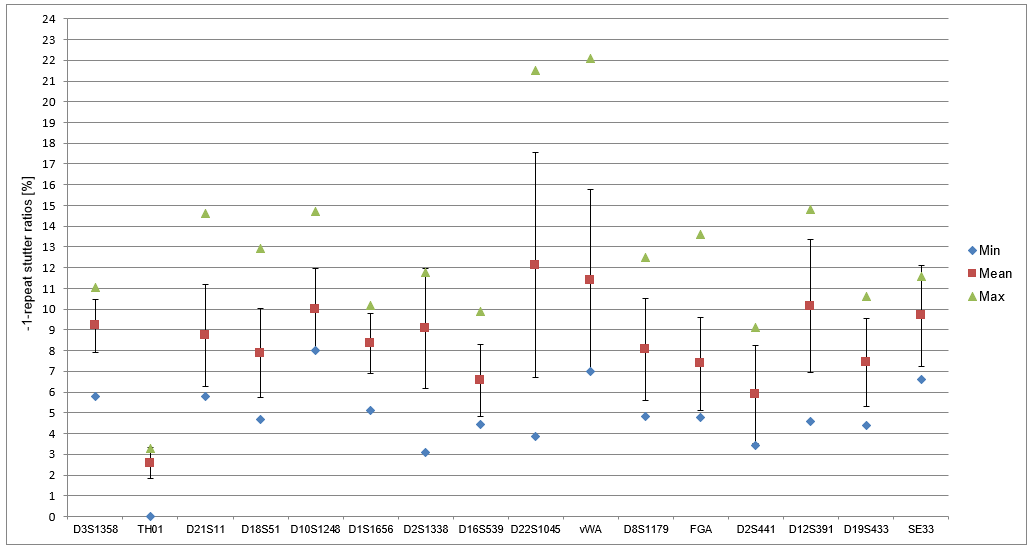


**Supplemental figure 5** -1-repeat stutter ratios [%] (minima, mean with SD and maxima) of the PowerPlex® ESX17 kit on the ABI PRISM® 310 Genetic Analyzer (n=10)


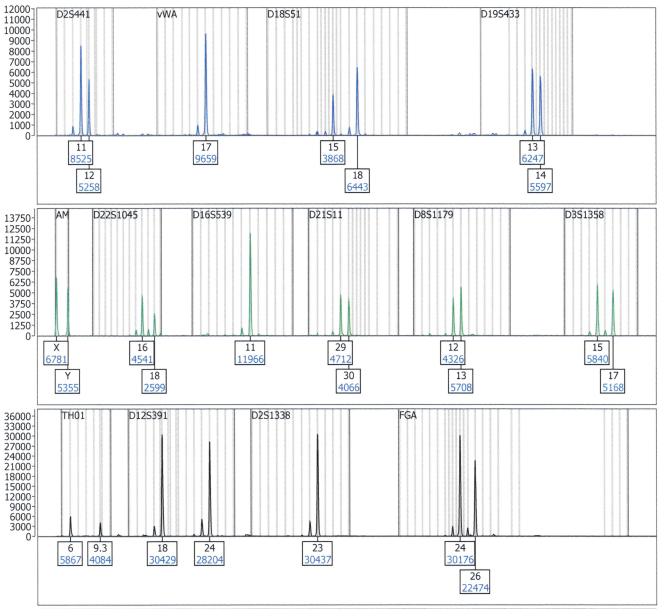

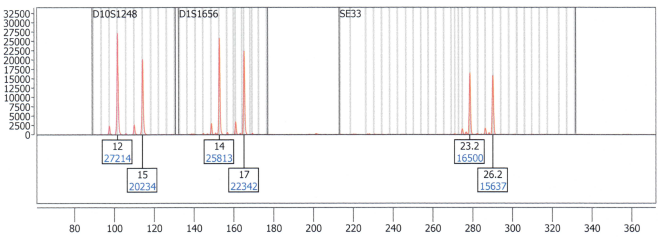


**Supplemental figure 6** Electropherogram of the positive control 9948 (Qiagen), amplified with the MPX5ESSv5 kit with 250 pg DNA template input in 12.5 µL reaction volume and analysed with the Spectrum Compact CE System and the GenoProof Mixture 2 software


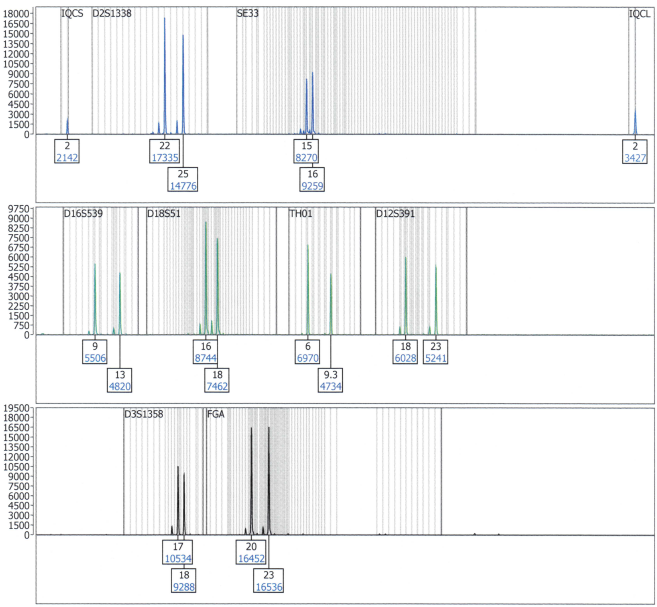

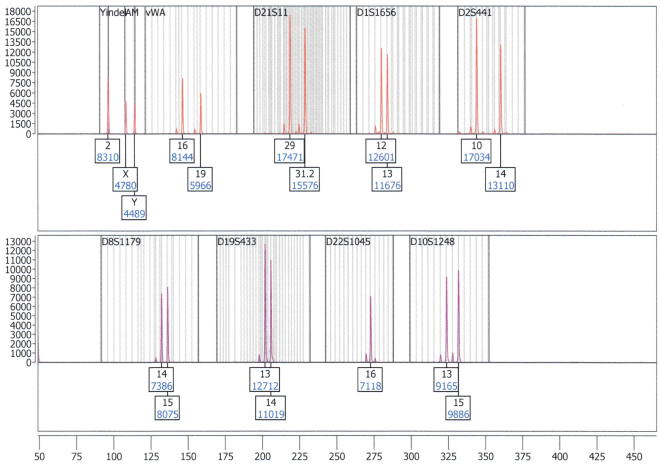


**Supplemental figure 7** Electropherogram of the positive control 2800M (Promega), amplified with the NGM Detect™ kit with 250 pg DNA template input in 12.5 µL reaction volume and analysed with the Spectrum Compact CE System and the GenoProof Mixture 2 software


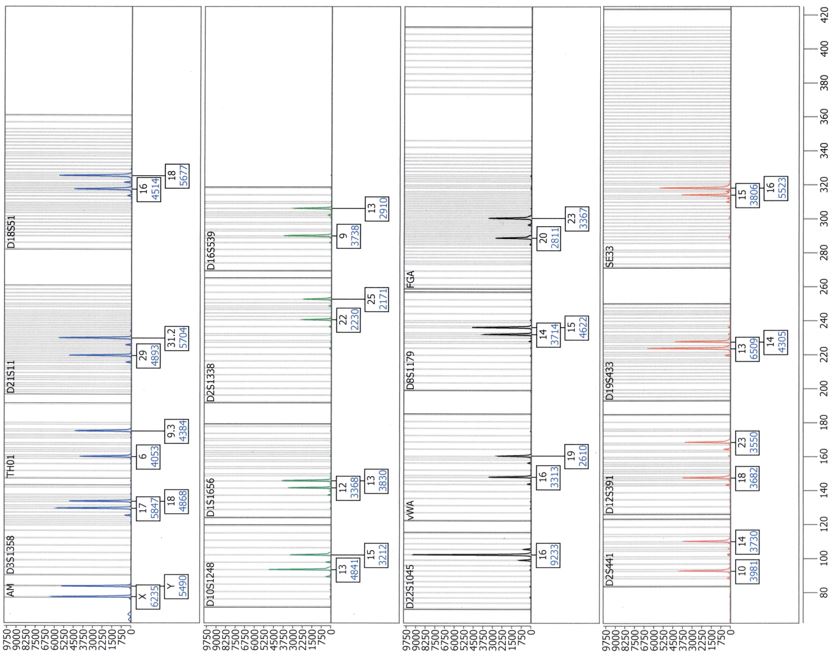


**Supplemental figure 8** Electropherogram of the positive control 2800M (Promega), amplified with the PowerPlex® ESX17 kit with 250 pg DNA template input in 12.5 µL reaction volume and analysed with the Spectrum Compact CE System and the GenoProof Mixture 2 software


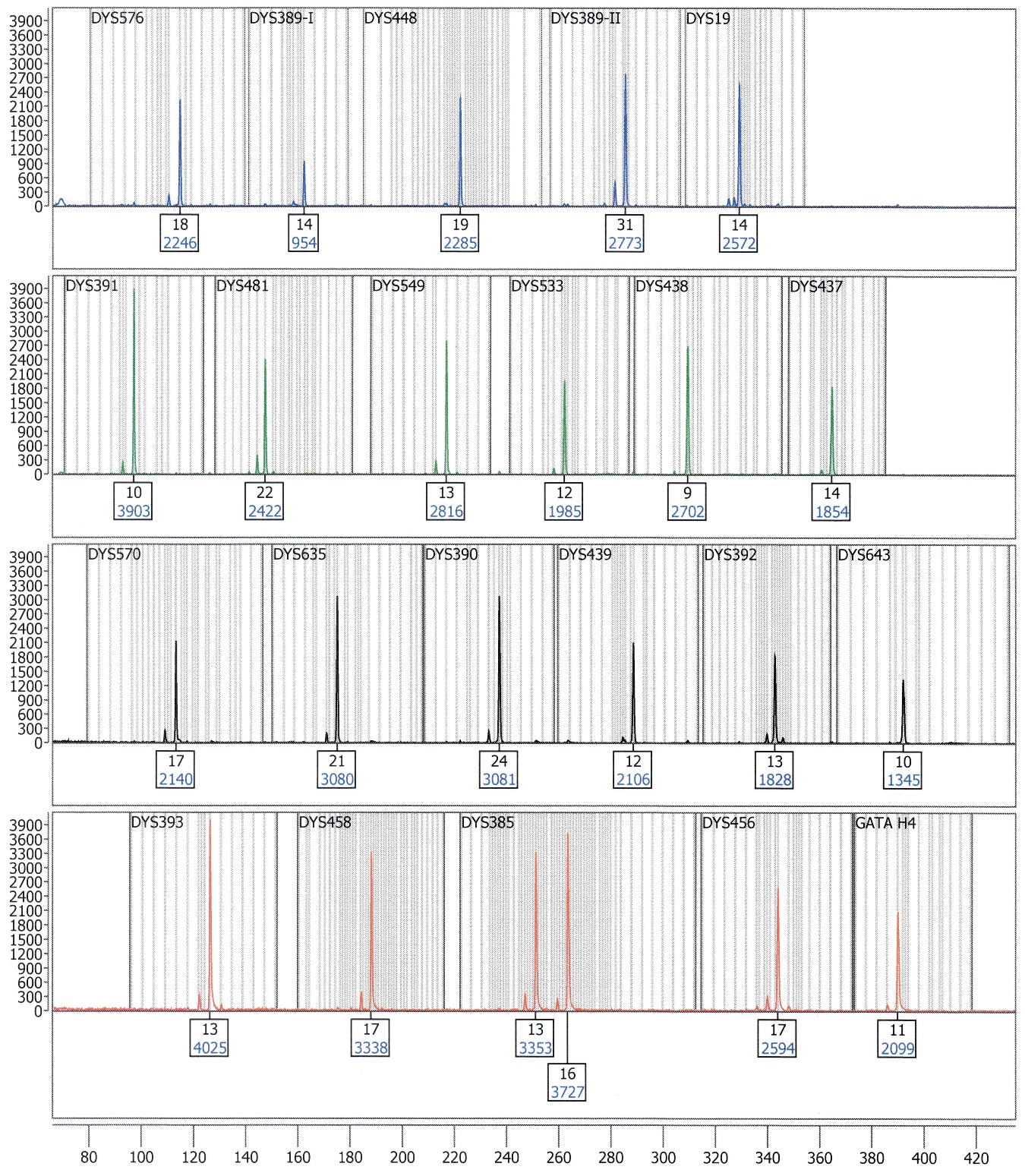


**Supplemental figure 9** Electropherogram of the positive control 2800M (Promega), amplified with the PowerPlex® Y23 kit with 250 pg DNA template input in 12.5 µL reaction volume and analysed with the Spectrum Compact CE System and the GenoProof Mixture 2 software


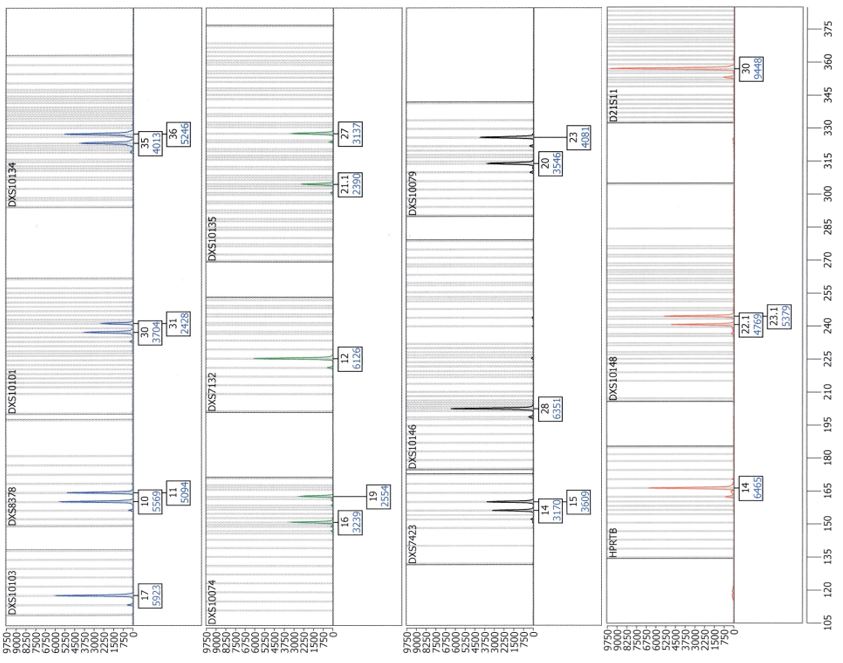


**Supplemental figure 10** Electropherogram of the positive control 9947A (Qiagen), amplified with the Investigator® Argus X-12 QS kit with 250 pg DNA template input in 12.5 µL reaction volume and analysed with the Spectrum Compact CE System and the GenoProof Mixture 2 software


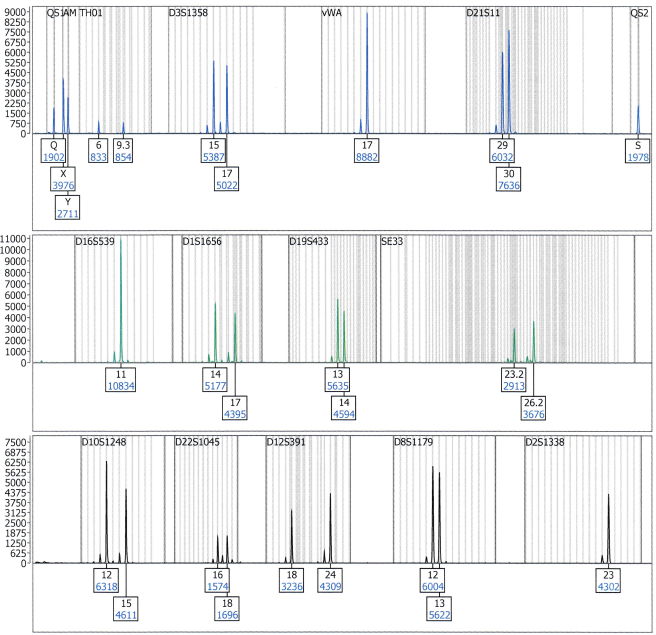

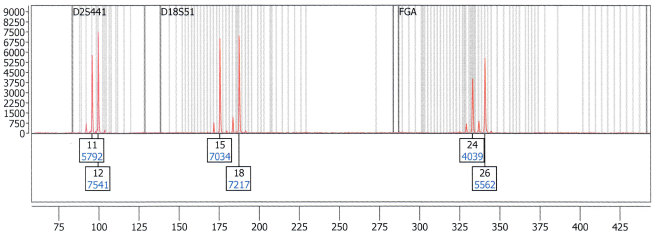


**Supplemental figure 11** Electropherogram of the positive control 9948 (Qiagen), amplified with the Investigator® ESSplex SE QS kit with 500 pg DNA template input in 25 µL reaction volume and analysed with the Spectrum Compact CE System and the GenoProof Mixture 2 software


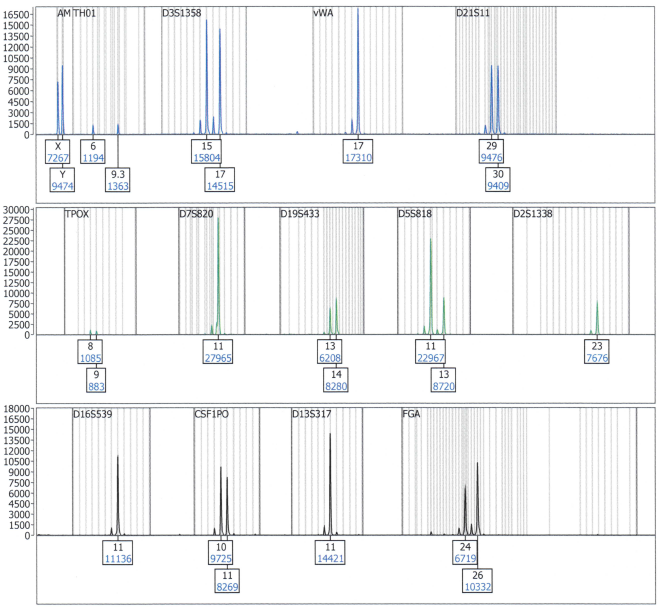

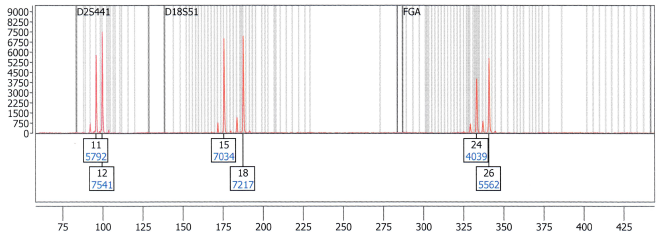


**Supplemental figure 12** Electropherogram of the positive control 9948 (Qiagen), amplified with the Investigator® IDPlex Plus kit with 500 pg DNA template input in 25 µL reaction volume and analysed with the Spectrum Compact CE System and the GenoProof Mixture 2 software


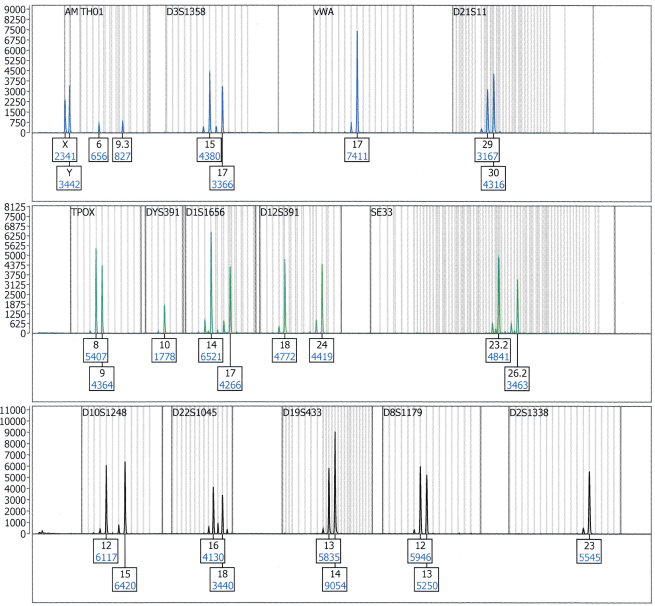

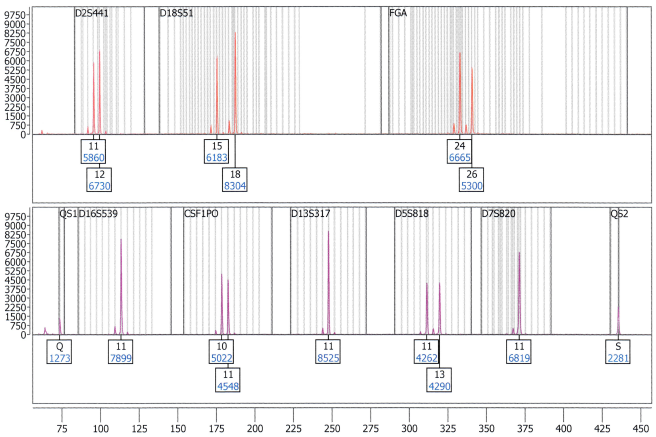


**Supplemental figure 13** Electropherogram of the positive control 9948 (Qiagen), amplified with the Investigator® 24Plex QS kit with 500 pg DNA template input in 25 µL reaction volume and analysed with the Spectrum Compact CE System and the GenoProof Mixture 2 software
